# Niacin supplementation induces type II to type I muscle fiber transition in skeletal muscle of sheep

**DOI:** 10.1186/1751-0147-55-85

**Published:** 2013-11-22

**Authors:** Muckta Khan, Aline Couturier, Johanna F Kubens, Erika Most, Frank-Christoph Mooren, Karsten Krüger, Robert Ringseis, Klaus Eder

**Affiliations:** 1 Institute of Animal Nutrition and Nutrition Physiology, Justus-Liebig-University Giessen, Heinrich-Buff-Ring 26-32, 35390 Giessen, Germany; 2 Department of Sports Medicine, Justus-Liebig-University Giessen, Kugelberg 62, 35394 Giessen, Germany

**Keywords:** Niacin, Sheep, Muscle fiber transition, Oxidative type I fiber

## Abstract

**Background:**

It was recently shown that niacin supplementation counteracts the obesity-induced muscle fiber transition from oxidative type I to glycolytic type II and increases the number of type I fibers in skeletal muscle of obese Zucker rats. These effects were likely mediated by the induction of key regulators of fiber transition, PPARδ (encoded by PPARD), PGC-1α (encoded by PPARGC1A) and PGC-1β (encoded by PPARGC1B), leading to type II to type I fiber transition and upregulation of genes involved in oxidative metabolism. The aim of the present study was to investigate whether niacin administration also influences fiber distribution and the metabolic phenotype of different muscles [*M. longissimus dorsi* (LD), *M. semimembranosus* (SM), *M. semitendinosus* (ST)] in sheep as a model for ruminants. For this purpose, 16 male, 11 wk old Rhoen sheep were randomly allocated to two groups of 8 sheep each administered either no (control group) or 1 g niacin per day (niacin group) for 4 wk.

**Results:**

After 4 wk, the percentage number of type I fibers in LD, SM and ST muscles was greater in the niacin group, whereas the percentage number of type II fibers was less in niacin group than in the control group (*P* < 0.05). The mRNA levels of PPARGC1A, PPARGC1B, and PPARD and the relative mRNA levels of genes involved in mitochondrial fatty acid uptake (CPT1B, SLC25A20), tricarboxylic acid cycle (SDHA), mitochondrial respiratory chain (COX5A, COX6A1), and angiogenesis (VEGFA) in LD, SM and ST muscles were greater (*P* < 0.05) or tended to be greater (*P* < 0.15) in the niacin group than in the control group.

**Conclusions:**

The study shows that niacin supplementation induces muscle fiber transition from type II to type I, and thereby an oxidative metabolic phenotype of skeletal muscle in sheep as a model for ruminants. The enhanced capacity of skeletal muscle to utilize fatty acids in ruminants might be particularly useful during metabolic states in which fatty acids are excessively mobilized from adipose tissue, such as during the early lactating period in high producing cows.

## Background

Pharmacological doses of niacin have long been known to lower the levels of blood lipids, especially triacylglycerols (TAG), but the mechanism underlying this effect is only incompletely understood. Even though it has been established that niacin inhibits lipolysis in adipocytes through binding to the niacin-receptor HCA_2_ and thereby reduces the supply of non-esterified fatty acids (NEFA) for hepatic TAG synthesis [1], this effect can only insufficiently explain the lipid-lowering effect because blood NEFA levels even become elevated during long-term niacin treatment due to a strong rebound phenomenon on lipolysis while the TAG lowering effect remains [2]. However, less well-documented niacin treatment also causes significant changes in gene expression in other tissues than adipose tissue, like skeletal muscle [2], a tissue which due to its great mass is particularly important for whole body fatty acid utilization. Noteworthy, it has been recently shown in humans that niacin administration induces the expression of two transcription factors, peroxisome proliferator-activated receptor δ (PPARδ, encoded by PPARD) and PPARγ coactivator-1α (PGC-1α, encoded by PPARGC1A) in skeletal muscle [3]. Both transcription factors are key regulators of muscle fiber composition and the muscle’s metabolic phenotype because they control genes involved in muscle fiber switching, fatty acid utilization, oxidative phosphorylation, mitochondrial biogenesis and function [4,5], and angiogenesis [6]. Skeletal muscle contains two major types of muscle fibers which differ in their contractile proteins and their metabolic capacity [7]. The type II fibers (“glycolytic fibers”) have a little number of mitochondria and largely generate ATP through glycolytic metabolism, whereas type I fibers (“oxidative fibers”) are mitochondria-rich and thus utilize mainly oxidative phosphorylation [8,9]. Interestingly, the distribution of type I and type II fibers of skeletal muscles shows high plasticity and can be altered by diverse factors, such as exercise, mechanical unloading, obesity or diabetes, resulting in a change of the muscle’s functional and metabolic phenotype [10-13]. In an attempt to study whether the induction of PPARδ and PGC-1α in skeletal muscle by pharmacological niacin doses leads to a change of muscle fiber distribution and the muscle’s metabolic phenotype, we have previously tested the effect of niacin supplementation at a dose used for reduction of serum lipids in obese Zucker rats [14] and pigs [15]. Both studies revealed that niacin supplementation induces muscle fiber transition from type II to type I and increases the number of type I fibers in skeletal muscle [14,15]. Moreover, we found that the expression of genes involved in fatty acid transport, mitochondrial fatty acid import and oxidation, oxidative phosphorylation and angiogenesis and genes encoding PPARδ, PGC-1α and PGC-1β (encoded by PPARGC1B), which, like PGC-1α, is a key regulator of skeletal muscle’s oxidative and contractile phenotype [16], in skeletal muscle is elevated by niacin treatment [14,15]. Thus, these findings suggest that niacin induces a change in the muscle metabolic phenotype which is indicative of an increased capacity of muscle for oxidative utilization of fatty acids and which might be useful during metabolic states where TAG and NEFA are strongly elevated, such as during early lactation in high producing dairy cows [17]. However, whether niacin treatment also causes type II to type I muscle fiber switching and increases the type I fiber content of skeletal muscles in ruminants has not been investigated yet. Thus, the present study aimed to investigate whether niacin administration at a pharmacological dose influences fiber distribution and the metabolic phenotype of different skeletal muscles in sheep as a model for ruminants. Niacin was administrated by drenching ensuring that the main part of the administrated niacin bypasses the rumen and reaches the small intestine.

## Methods

### Animals, housing, and experimental design

The experiment was located at the Research Station of the Institute of Animal Breeding and Genetics at the University of Giessen, Germany. A total of 16 male, 11 wk old Rhoen sheep with an average body weight of 29.6 ± 3.0 (mean ± SD) kg were randomly allocated to two groups of 8 sheep each (control group and niacin group). All sheep within one group were kept together in a barn on straw. All sheep received hay *ad libitum* and 1.5 kg concentrate per day and sheep. The hay contained (% of dry matter) 47.5% nitrogen-free extractable substances, 30.3% crude fiber, 7.0% crude protein, 6.1% crude ash and 1.1% crude fat. The concentrate (RWZ-Schaf 18 Uni Press, RWZ, Köln) consisted of (g/kg): Root pulp (250), wheat (200), dried distillers grains with solubles (120), wheat bran (104), wheat gluten feed (100), rapeseed extraction meal (100), soybean extraction meal (37), calcium carbonate (22), soy hulls (20), molasses (20), vinasse (10), monocalcium phosphate (8), sodium chloride (1.9), magnesium oxide (1.6) and a premix supplying vitamins and minerals (5.5; amounts of vitamins and minerals supplied per kg: vitamin A, 8,000 IE; vitamin D3, 1,000 IE; vitamin E, 65 mg; zinc, 40 mg as zinc sulfate monohydrate; manganese, 20 mg as manganese (II) sulfate monohydrate; selenium, 0.2 mg as sodium selenite; cobalt, 0.2 mg as cobalt (II) sulfate monohydrate; iodine, 0.1 mg as calcium iodate). According to the manufacturer’s declaration the concentrate contained 10.6 MJ ME/kg and 18% crude protein. Additionally, sheep of the niacin group received 1 g niacin (obtained from Lonza, Basel, Switzerland) dissolved in 100 ml drinking water by drenching daily at eleven a.m. Sheep of the control group were given the same amount of drinking water by drenching without addition of niacin. Since the concentrate did not contain any supplemental niacin, the sheep of the control group received only the niacin contained in the hay and the feed components of the concentrate, from which no actual concentrations of niacin are available. Based on literature data, the niacin concentration in hay and concentrate is below 100 mg/kg dry matter [18]. The experimental period during which sheep were administered either no (control group) or 1 g niacin per day (niacin group) lasted for 4 wk. Water was given *ad libitum*. All experimental procedures were in strict accordance with the recommendations in the guidelines for the care and use of laboratory animals [19] and the Appendix A of European Convention for the Protection of Vertebrate Animals used for Experimental and other Scientific Purposes. In accordance with article 4 par. 3 of the German Animal Welfare Law all animals were humanely killed for scientific purpose approved by the Animal Welfare Officer of the Justus-Liebig-University.

### Sample collection

After 4 wk the animals were slaughtered at a commercial slaughterhouse located in the near of the Research Station. Blood samples were taken into EDTA polyethylene tubes (Sarstedt, Nürnbrecht, Germany) and plasma was collected by centrifugation (1,100 × g; 10 min, 4°C). Samples from three different skeletal muscles [*M. longissimus dorsi* (LD), *M. semimembranosus* (SM), *M. semitendinosus* (ST)] were excised nearly at the same location and samples were shock frozen with liquid nitrogen and stored at −80°C pending analysis.

### Muscle fiber typing

Fiber typing was performed as recently described in detail [14]. In brief, 30 μm thick, serial cross sections were taken using a cryostat microtome, mounted on cover slips and stained for myosin ATPase (mATPase) using a modified method of Hämäläinen and Pette [20]. In brief, sections were pre-incubated for 5 min in sodium acetate (54.3 mM) – sodium barbital (32.6 mM) solution adjusted with hydrogen chloride to pH 4.6. After washing, the sections were incubated for 30 min at 37°C in substrate solution (2.7 mM ATP, 100 mM glycin, 54 mM calcium (II) chloride, 100 mM sodium chloride, pH adjusted to 9.6). Following incubation in 1% calcium (II) chloride and 2% cobalt (II) chloride, a black insoluble compound was developed in 1% ammonium sulfide for 50 s leading to a black staining of type I fibers and grey staining of type II fibers. Subsequently, the sections were analyzed by light microscopy (Leica DMI 6000B) for calculating the type I and type II fiber percentages. Fiber typing was carried out in the best five images out of ten stained sections per muscle and animal, and all fibers within a 100 cm^2^ area were calculated. This area corresponded to about 60 fibers. Thus, a total of 300 fibers were calculated per animal and muscle.

### Determination of nicotinic acid and nicotinamide concentrations in plasma

Concentrations of nicotinic acid and nicotineamide in plasma were determined by LC-MS/MS according to the method from Liu et al. [21].

### Determination of plasma lipids

The plasma concentrations of TAG and NEFA were measured using enzymatic reagent kits from Merck Eurolab (ref. 113009990314) and from Wako Chemicals (ref. RD291001200R), respectively.

### RNA isolation and qPCR analysis

RNA isolation, cDNA synthesis qPCR analysis were performed as described recently in detail [22]. In brief, total RNA was isolated from 25–30 mg skeletal muscle aliquots using Trizol™ reagent (Invitrogen, Karlsruhe, Germany), and RNA concentration and purity were estimated from the optical density at 260 and 280 nm (Infinite 200 M microplate reader, Tecan, Männedorf, Switzerland). RNA integrity was assessed by confirming intact bands corresponding to the 18S and 28S ribosomal RNA subunits using 1% agarose gel electrophoresis. Following cDNA synthesis within one week after RNA isolation using dT18 primer and M-MuLV Reverse Transcriptase (MBI Fermentas, St. Leon-Rot, Germany), qPCR analysis was performed as described recently in detail [22]. Features of gene-specific primer pairs are listed in Table 1. Calculation of gene expression data and normalization by GeNorm normalization factor were carried out as described recently [22]. The normalization factor was calculated as the geometric mean of expression data of the three most stable out of six tested potential reference genes (RPL19, YWHAZ, RPS26, MDH1, B2M, and GAPDH). In each muscle the three most stable reference genes were the same (the stability score M as calculated by GeNorm is shown in brackets): LD muscle: RPL19 (0.025), YWHAZ (0.026), and RPS26 (0.029); SM muscle: RPL19 (0.026), YWHAZ (0.028), and RPS26 (0.028); ST muscle: RPL19 (0.033), YWHAZ (0.037), and RPS26 (0.040). Means and SD were calculated from normalized expression data for samples of the same treatment group. The mean of the group control was set to 1 and mean and SD of the niacin group were scaled proportionally. Data on qPCR performance for target and reference genes measured in skeletal muscle are shown in Table 2.

**Table 1:** Characteristics of primers used for qPCR

**Gene**	**Forward primer (3′-5′)**	**Reverse primer (5′-3′)**	**Product length (bp)**	**T**_**m **_**(°C)**	**NCBI Genbank**
*Reference genes*					
B2M	GCGTATTCCAGAGGTCCAGG	CGGCAGCTGTACTGATCCTT	234	60	NM_001009284
GAPDH	GGCGTGAACCACGAGAAGTA	GCAGGGATGATGTTTTGGGC	227	60	AF022183
MDH1	TACGTGTTCCCTGGAGTTGC	TGCTTCCTTGTTTGGGGGTT	249	57	NM_001135220
RPL19	AGCCTGTGACTGTCCATTCC	TTCTCGGGCATTCGAGCATT	118	57	JN811679
RPS26	ACAACGGTCGTGCCAAAAAG	AAATCGGGGTGGAGGTGTTC	284	57	NM_001009435
YWHAZ	AGACGGAAGGTGCTGAGAAA	TGGGGATCAAGAACTTTTCCAA	120	57	JN811681
*Target genes*					
COX5A	GCTCGCTGGGTGACATACTT	ACCTCTAGGATGCGAACTGC	173	60	AF233074
COX6A1	TGCAGCTGAGTCGGTGTATG	GAACTCGGGTCTCTCCTCCT	161	60	GU585577
CPT1B	GACGTTTCCATGGGACTGGT	GCCAGCGTCTCCATTCGATA	389	60	NM_001009259
MHCI	TCGTCAAGGCCACAATTTTG	CTGTCGCAACACCTGGTCCT	100	60	AB058898
MHCIIA	AAGCCTTTTGATGCCAAGACAT	TTCACCGTCACTTTCCCACC	100	60	AB058896
MHCIIX	CTTCGTGGCGGACCCTAAG	CAGTTACTGTCGCCCCAGCT	100	60	AB058897
PPARD	TCAGCGTGCACGTCTTCTAC	CAGGAATTCCCGGGTGACAA	230	59	XM_004018769
PPARGC1A	GGTGACCATGACTATTGTCAG	CTCGGATTTCCTGGTCTTGAA	216	58	XM_004009738
PPARGC1B	CTGGACCGAGTTCTCCATCC	CACGTGCCCTTTCACCTGCA	244	61	XM_004008965
SDHA	GTTTGAGCAGCACTGGAGGA	AGTCGGTCTCGTTCAAAGTCC	110	60	DQ386895
SLC25A20	CCGAGGGATCTACAAGGGGA	CCTTCATCCCGGATCAGCTC	288	61	NM_001127277
VEGFA	GGACATCTTCCAGGAGTACC	GCATGGTGATGTTGAACTCCT	137	58	EU857623

**Table 2:** qPCR performance data

**Gene**	**Slope**	**R**^**2#**^	Efficiency*
B2M	−3.20	0.999	2.05
COX5A	−3.22	0.999	2.04
COX6A1	−2.99	0.997	2.16
CPT1B	−3.79	0.996	1.84
GAPDH	−2.97	0.999	2.17
MDH1	−3.21	0.999	2.05
MHCI	−3.37	1.000	1.98
MHCIIA	−3.21	0.998	2.05
MHCIIX	−3.29	0.999	2.01
PPARD	−3.05	0.967	2.13
PPARGC1A	−3.34	0.999	1.99
PPARGC1B	−3.29	0.956	2.01
RPL19	−3.31	0.997	2.00
RPS26	−3.72	0.998	1.86
SDHA	−3.12	0.999	2.09
SLC25A20	−3.81	0.980	1.83
VEGFA	−3.31	0.997	2.00
YWHAZ	−3.34	0.993	1.99

^#^Coefficient of determination of the standard curve.

*The efficiency is determined by [10^(−1/-slope^].

### Immunoblotting

Preparation of homogenates, determination of protein concentration and immunoblotting were performed as described recently in detail [23]. In brief, proteins were separated by 12,5% SDS-PAGE, transferred to a nitrocellulose membrane and incubated with primary antibodies against PGC-1α (dilution 1:1000; polyclonal anti-PGC-1α antibody; Millipore, Temecula, CA), PPARδ (dilution 1:1000; polyclonal anti-PGC-1α antibody; Abcam, Cambridge, UK), and glyceraldehyde-3-phosphate dehydrogenase (GAPDH) (dilution 1:5000; monoclonal anti-GAPDH antibody, Abcam, Cambridge, UK) as a reference protein. Nitrocellulose membranes were washed, and subsequently incubated with a horseradish peroxidase conjugated secondary monoclonal anti-mouse-IgG antibody (Abcam, Cambridge, UK) for GAPDH and polyclonal anti-rabbit-IgG antibody (Sigma-Aldrich, St. Louis, Germany) for PGC-1α, and PPARδ at room temperature. Finally, blots were developed by either ECL Select or ECL Prime (both GE Healthcare, Munich, Germany), respectively, and the intensities of the specific bands detected with a Bio-Imaging system (Syngene, Cambridge, UK) and quantified by Syngene GeneTools software (nonlinear dynamics).

### Statistics

Data were statistically analysed by Student’s t-test using the Minitab Statistical Software (Rel. 13.0, State College, PA, USA). Means were considered significantly different for *P* < 0.05. Data presented are shown as means ± SD.

## Results

### Final body weight, body weight gain and carcass weight

Final body weights, daily body weight gain and carcass weights did not differ between the control group and the niacin group (Final body weight: 37.4 ± 2.3 vs. 37.8 ± 3.7 kg; daily body weight gain: 308 ± 50 vs. 308 ± 41 g; carcass weight: 17.2 ± 1.4 vs. 17.3 ± 2.4 kg; control group vs. niacin group; n = 8/group).

### Concentrations of nicotinic acid and its metabolite nicotinamide in plasma

The plasma concentrations of nicotinic acid and its metabolite nicotinamide were greater in the niacin group than in the control group (nicotinic acid: 0.41 ± 0.31 vs. 0.75 ± 0.42 μg/mL; nicotinamide: 0.46 ± 0.25 vs. 3.42 ± 0.90 μg/mL; control group vs. niacin group; *P* < 0.05).

### Lipid concentrations in plasma

In order to assess the lipid-lowering properties of niacin in sheep, we determined the plasma concentrations of NEFA and TAG. The plasma TAG concentration tended to be lower in the niacin group than in the control group (0.20 ± 0.02 vs. 0.17 ± 0.03 mmol/L; control group vs. niacin group; *P* < 0.1). The plasma NEFA concentration did not differ between the niacin and the control group (0.32 ± 0.11 vs. 0.29 ± 0.14 mmol/L; control group vs. niacin group).

### Muscle fiber type composition and expression of fiber-specific myosin heavy chain (MHC) isoforms in skeletal muscles

**Figure 1:**
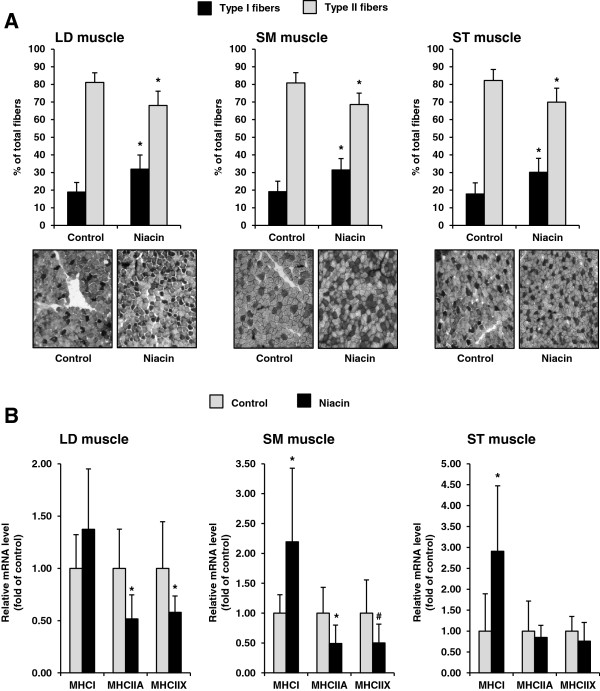
**Muscle fiber type distribution (A), and relative mRNA levels of fiber-specific MHC isoforms (B) of LD muscle, SM muscle, and ST muscle of sheep administered either no (control group) or 1 g niacin per day (niacin group) for 4 wk.** Bars represent means ± SD, n = 8 sheep/group. Representative images from fiber typing for each group are shown below the graph showing fiber type distribution, with the “black” areas being the type I fibers and the “grey” ones being the type II fibers. *different from control group, *P* < 0.05, ^#^different from control group, *P* < 0.15.

To evaluate an effect of niacin on fiber type distribution, muscle fiber typing and transcript level measurement of fiber-specific MHC isoforms was carried out. As shown in Figure 1A, the percentage number of type I fibers in LD muscle, SM muscle and ST muscle was greater in the niacin group than in the control group, whereas the percentage number of type II fibers was less in niacin group than in the control group (*P* < 0.05). The PCR primers used to detect transcript levels of sheep MHC isoforms corresponding to MHCI, MHCIIA and MHCIIX have been designed based on available sheep partial-length cDNA sequences [24]. Specific PCR primers for sheep MHCIIB transcripts could not be designed because no sheep MHCIIB cDNA sequence is available in nucleic acid databases. As shown in Figure 1B, the mRNA level of type I-specific MHCI in SM muscle and ST muscle was increased in the niacin group compared to the control group (*P* < 0.05). In LD muscle, the mRNA level of MHCI was numerically greater in the niacin group relative to the control group (*P* = 0.24). The mRNA level of MHCIIA was decreased in LD muscle and SM muscle of the niacin group compared to the control group (*P* < 0.05), but did not differ between groups in ST muscle. The mRNA level of MHCIIX in LD muscle and SM muscle was significantly less (*P* < 0.05) and tended to be less (*P* < 0.15), respectively, in the niacin group than in the control group. In ST muscle, the mRNA level of MHCIIX was not different between groups.

### Expression of key regulators of muscle fiber transition in skeletal muscles

**Figure 2:**
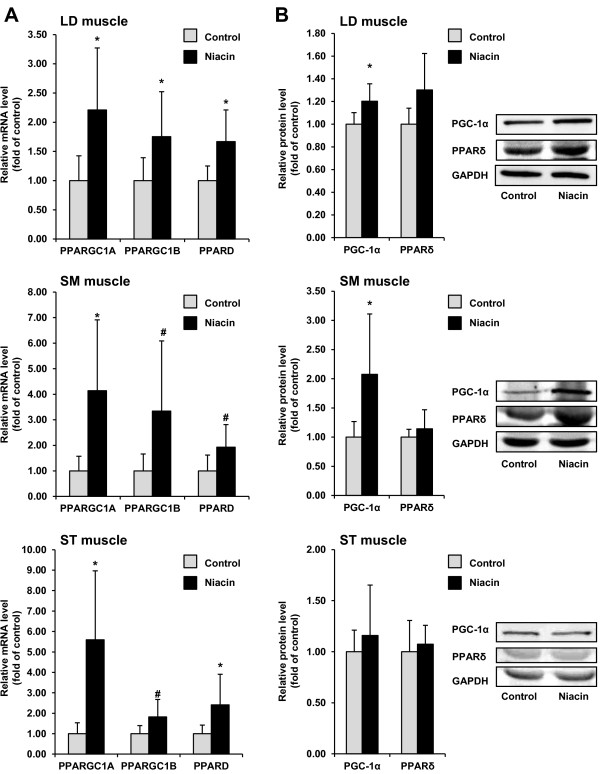
**Relative mRNA levels of PPARGC1A, PPARGC1B and PPARD (A), and relative protein levels of PGC-1α and PPARδ (B) in LD muscle, SM muscle, and ST muscle of sheep administered either no (control group) or 1 g niacin per day (niacin group) for 4 wk.** Bars represent means ± SD, n = 8 (mRNA) and 6 (protein) sheep/group. Representative immunoblots specific to PGC-1α, PPARδ and GAPDH as internal control are shown for one animal per group; immunoblots for the other animals revealed similar results. *different from control group, *P* < 0.05, ^#^different from control group, *P* < 0.15.

To explore the mechanisms underlying the niacin-induced muscle fiber transition we determined mRNA and/or protein levels of the key regulators of muscle fiber transition, PGC-1α, PGC-1β and PPARδ, in the three muscles. The mRNA level of PPARGC1A in all three muscles was greater in the niacin group than in the control group (*P* < 0.05; Figure 2). The mRNA level of PPARGC1B was greater in LD muscle (*P* < 0.05) and tended to be greater in SM muscle and ST muscle (*P* < 0.15) of the niacin group than in the control group (Figure 2). The mRNA level of PPARD was increased in LD muscle and ST muscle (*P* < 0.05) and tended to be increased in SM muscle (*P* < 0.15; Figure 2). The protein level of PGC-1α was elevated in LD muscle and SM muscle of the niacin group compared to the control group (*P* < 0.05), but did not differ in ST muscle between groups (Figure 2). The protein level of PPARδ in all three muscles did not differ between groups.

### Expression of genes involved in fatty acid oxidation, mitochondrial respiratory chain and angiogenesis in skeletal muscles

**Figure 3:**
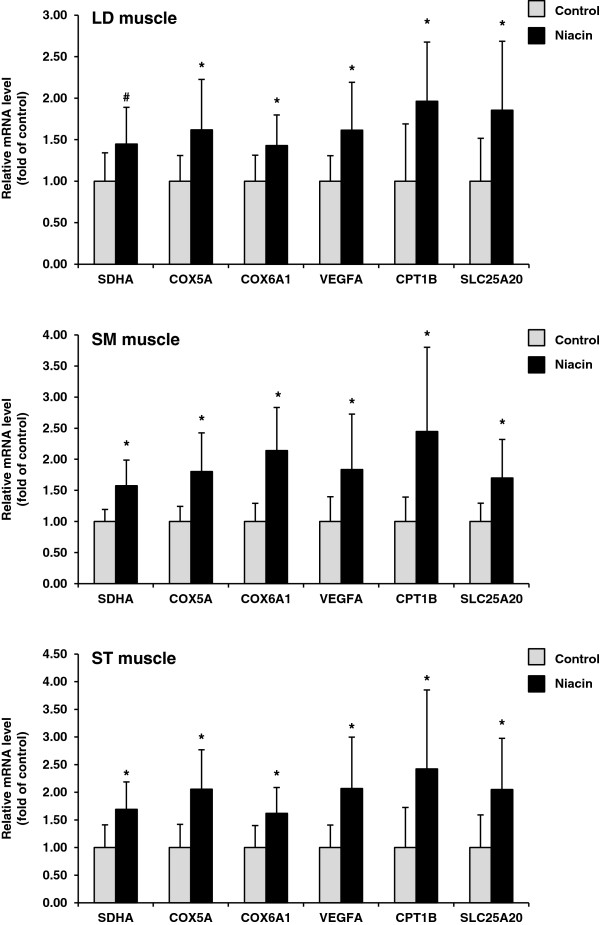
**Relative mRNA levels of SDHA, COX5A, COX6A1, VEGFA, CPT1B, and SLC25A20 in LD muscle, SM muscle and ST muscle of sheep administered either no (control group) or 1 g niacin per day (niacin group) for 4 wk.** Bars represent means ± SD for n = 8 sheep/group. *different from control group, *P* < 0.05; ^#^different from control group, *P* < 0.15.

Since PGC-1α and PPARδ are important regulators of genes involved in fatty acid oxidation, mitochondrial respiratory chain and angiogenesis, we determined mRNA levels of CPT1B and SLC25A20, which encode two enzymes of the carnitine shuttle system, SDHA, which encodes the tricarboxylic acid cycle (TCA) enzyme succinate dehydrogenase, COX6A1 and COX5A, which encode two subunits of the respiratory chain complex IV (cytochrome c oxidase), and VEGFA encoding the angiogenic factor VEGF-a. Relative mRNA levels of COX5A, COX6A1, VEGFA, CPT1B, and SLC25A20 in all three muscles were greater in the niacin group than in the control group (*P* < 0.05; Figure 3). In addition, the relative mRNA level of SDHA in SM muscle and ST muscle was greater in the niacin group than in the control group (*P* < 0.05; Figure 3). In LD muscle, the mRNA level of SDHA tended to be greater in the niacin group than in the control group (*P* < 0.15; Figure 3).

## Discussion

In the present study we tested the hypothesis that, like in rats and pigs [14,15], niacin supplementation induces muscle fiber transition from type II (glycolytic) to type I (oxidative), and thereby an oxidative metabolic phenotype of skeletal muscle in sheep as a ruminant model. The dietary niacin dosage (1 g niacin per day) given to the sheep related to 27–35 mg/kg body weight which is only slightly below that given to the rats (40–54 mg/kg body weight [14]) and pigs (30–49 mg/kg body weight [15]) in our recent studies and which was shown to induce a muscle fiber switch from type II to type I in skeletal muscle. The niacin dosage administered by drenching to the sheep of the niacin group was markedly higher than that taken up from the feed ration (hay and concentrate) by the sheep of the control group, because according to literature data the native concentration of niacin in hay and the main components of the concentrate is below 100 mg/kg dry matter [18]. In line with this, the niacin administration to the sheep caused a significant increase in the plasma concentration of the nicotinic acid metabolite nicotinamide. In addition, it has to be considered that the sheep used in this study had already fully developed rumen. This means that the niacin requirement for the sheep was covered from niacin synthesized by the rumen microbes and that the niacin from the ingested hay and concentrate was largely degraded by rumen microbes [25]. In contrast, the drenching procedure, which was used to administer the daily niacin bolus, is a suitable approach to ensure that the main part of the administered niacin bypasses the rumen and reaches the small intestine. In the present study, we considered three different skeletal muscles, LD, SM and ST, containing predominantly type II fibers (the type II fiber percentage in all three muscles in the control group was approximately 81%), because we expected an effect of niacin only in skeletal muscles with a high percentage of type II fibers. The main finding of the present study is that supplementation of niacin induces muscle fiber switching also in skeletal muscles of sheep. Muscle fiber typing revealed that the type I fiber percentage in the three muscles investigated increased from approximately 18–20% in the control group to 30–31% in the niacin group, whereas the type II fiber percentage decreased from 81% to 69%. In line with this, we observed that the mRNA level of the type I-specific MHCI was significantly greater in SM muscle and ST muscle and tended to be greater in LD muscle, but the mRNA levels of type II-specific MHC isoforms in LD and SM muscle were less in the niacin group than in the control group.

Regarding that muscle fiber transition is induced on the molecular level by an increased activity of PGC-1α, PGC-1β and PPARδ [4,5,26,27], we determined the mRNA and/or protein levels of these key regulators in the three muscles. We found that the mRNA level of PPARGGC1A in all three muscles was markedly elevated, and the mRNA levels of PPARGC1B and PPARD in all three muscles were either significantly increased or tended to be increased in the niacin group compared to the control group. In addition, the protein level of PGC-1α in two of three muscles was greater in the niacin than in the control group, whereas the protein level of PPARδ in all muscles was not different between groups. The PGC-1β protein level could not be determined, because no appropriate antibody to reliably detect PGC-1β was available. We cannot definitely explain the lack of effect of niacin on PPARδ protein levels, but this may be due to the comparatively small sensitivity of the western blotting technique making it difficult to detect slight differences between groups. However, the unaltered protein level of PPARδ does not exclude that its DNA-binding activity was increased because it is known that PGC-1α and PGC-1β, whose genes expression was clearly increased, act as coactivators of PPARδ and enhance the transactivation activity of PPARδ [28]. Therefore, our finding suggests that niacin supplementation increases the transcriptional activity of these critical regulators of muscle fiber transition, and thus provides an explanation for the increased type I fiber content in skeletal muscles of niacin-treated sheep.

Type I fibers, also called slow-twitch oxidative fibers, contain a high number of mitochondria, have a high oxidative capacity, and preferentially use fatty acids for energy production [8,9]. This oxidative metabolic phenotype of type I fibers is the consequence of a markedly higher expression of genes involved in fatty acid transport and uptake, β-oxidation, carnitine shuttle, TCA cycle and respiratory chain compared to glycolytic type II fibers [26,27]. In addition, type I fibers exhibit a higher expression of angiogenic factors, like VEGFA, which favors the preferential use of fatty acids by type I fibers because angiogenic factors increase capillary density and thereby blood perfusion but also the expression of fatty acid transport proteins [29]. In the present study we could demonstrate that several genes encoding proteins involved in oxidative metabolism (SDHA, COX5A, COX6A1, VEGFA, CPT1B, SLC25A20) were up-regulated in the muscles of the niacin group compared to the control group which is in line with the niacin-induced changes in fiber type distribution and expression of MHC isoforms. Although we did not provide data showing that the increased expression of oxidative genes is also accompanied by an enhanced activity of the encoded enzymes and an elevated capillary density, we suggest that the niacin-induced changes in skeletal muscle mRNA levels are indicative of an improved oxidative capacity because it is well known that the changes in the muscle’s metabolic and contractile phenotype are induced at the transcriptional level through an enhanced activity of PGC-1α and PPARδ [26,27].

## Conclusions

The results of this study show that niacin supplementation in sheep as a model for ruminants induces muscle fiber transition from type II (glycolytic) to type I (oxidative) being indicative of a change of the muscle’s metabolic phenotype towards a more oxidative one. An enhanced capacity of skeletal muscle to utilize fatty acids in ruminants might be particularly useful during metabolic states in which fatty acids are extensively mobilized from adipose tissue, such as during the early lactating period in high producing cows. In addition, considering that several studies have reported that oxidative muscles with a high percentage of type I fibers have a lower glycolytic potential, a darker color and a higher ultimate pH [30-32], the niacin-induced change in the muscle’s fiber type distribution may influence meat quality. At least in pigs it was demonstrated that oxidative muscle types tend to develop dark, firm and dry pork in response to intense physical activity and/or high psychological stress levels preslaughter [33]. Thus future studies have to investigate whether niacin administration influences meat quality from sheep.

## Competing interests

The authors declare that they have no competing interests.

## Authors’ contributions

MK conducted the animal experiment, performed fiber typing, PCR analyses, blood lipid analyses and statistical analyses, and wrote the manuscript. AC and JFK performed immunoblotting. EM performed nicotinic acid and nicotinamide determination in blood. FCM and KK analysed data from muscle fiber typing. RR supervised PCR analyses, immunoblotting and statistical analysis and helped to draft the manuscript. KE conceived of the study, participated in its design and coordination and helped to draft the manuscript. All authors read and approved the final manuscript.

## References

[B1] Gille A Bodor ET Ahmed K Offermanns S Nicotinic acid: pharmacological effects and mechanisms and mechanisms of action Annu Rev Pharmacol Toxicol 2008 48 79 106 10.1146/annurev.pharmtox.48.113006.094746 17705685

[B2] Choi S Yoon H Oh KS Oh YT Kim YI Kang I Youn JH Widespread effects of nicotinic acid on gene expression in insulin-sensitive tissues: implications for unwanted effects of nicotinic acid treatment Metabolism 2011 60 134 144 10.1016/j.metabol.2010.02.013 20303128 PMC2912158

[B3] Watt MJ Southgate RJ Holmes AG Febbraio MA Suppression of plasma free fatty acids upregulates peroxisome proliferator-activated receptor (PPAR) α and δ and PPAR coactivator 1α in human skeletal muscle, but not lipid regulatory genes J Mol Endocrinol 2004 33 533 544 10.1677/jme.1.01499 15525607

[B4] Wang YX Zhang CL Yu RT Cho HK Nelson MC Bayuga-Ocampo CR Ham J Kang H Evans RM Regulation of muscle fiber type and running endurance by PPARδ PLoS Biol 2004 2 e294 10.1371/journal.pbio.0020294 15328533 PMC509410

[B5] Schuler M Ali F Chambon C Duteil D Bornert JM Tardivel A Desvergne B Wahli W Chambon P Metzger D PGC1α expression is controlled in skeletal muscles by PPAR β, whose ablation results in fiber-type switching, obesity, and type 2 diabetes Cell Metab 2006 4 407 414 10.1016/j.cmet.2006.10.003 17084713

[B6] Chinsomboon J Ruas J Gupta RK Thom R Shoag J Rowe GC Sawada N Raghuram S Arany Z The transcriptional coactivator PGC-1alpha mediates exercise-induced angiogenesis in skeletal muscle Proc Natl Acad Sci USA 2009 106 21401 21406 10.1073/pnas.0909131106 19966219 PMC2795492

[B7] Pette D Staron RS Cellular and molecular diversities of mammalian skeletal muscle fibers Rev Physiol Biochem Pharmacol 1990 116 1 76 2149884 10.1007/3540528806_3

[B8] Peter JB Barnard RJ Edgerton VR Gillespie CA Stempel KE Metabolic profiles of three fiber types of skeletal muscle in guinea pigs and rabbits Biochemistry 1972 11 2627 2633 10.1021/bi00764a013 4261555

[B9] Barnard RJ Edgerton VR Furukawa T Peter JB Histochemical, biochemical, and contractile properties of red, white, and intermediate fibers Am J Physiol 1971 220 410 414 4250606 10.1152/ajplegacy.1971.220.2.410

[B10] Waters RE Rotevatn S Li P Annex BH Yan Z Voluntary running induces fiber type-specific angiogenesis in mouse skeletal muscle Am J Physiol Cell Physiol 2004 287 C1342 1348 10.1152/ajpcell.00247.2004 15253894

[B11] Cassano P Sciancalepore AG Pesce V Flück M Hoppeler H Calvani M Mosconi L Cantatore P Gadaleta MN Acetyl-L-carnitine feeding to unloaded rats triggers in soleus muscle the coordinated expression of genes involved in mitochondrial biogenesis Biochim Biophys Acta 2006 1757 1421 1428 10.1016/j.bbabio.2006.05.019 16814248

[B12] Fujita N Nagatomo F Murakami S Kondo H Ishihara A Fujino H Effects of hyperbaric oxygen on metabolic capacity of the skeletal muscle in type 2 diabetic rats with obesity Scientific World Journal 2012 2012 637978 22778702 10.1100/2012/637978 PMC3385605

[B13] Nagatomo F Fujino H Kondo H Gu N Takeda I Ishioka N Tsuda K Ishihara A PGC-1α mRNA level and oxidative capacity of the plantaris muscle in rats with metabolic syndrome, hypertension, and type 2 diabetes Acta Histochem Cytochem 2011 44 73 80 10.1267/ahc.10041 21614168 PMC3096084

[B14] Ringseis R Rosenbaum S Gessner DK Herges L Kubens JF Mooren FC Krüger K Eder K Supplementing obese Zucker rats with niacin induces the transition of glycolytic to oxidative skeletal muscle fibers J Nutr 2013 143 125 131 10.3945/jn.112.164038 23256146

[B15] Khan M Ringseis R Mooren FC Krüger K Most E Eder K Niacin supplementation increases the number of oxidative type I fibers in skeletal muscle of growing pigs BMC Vet Res 2013 9 177 10.1186/1746-6148-9-177 24010567 PMC3846775

[B16] Arany Z Lebrasseur N Morris C Smith E Yang W Ma Y Chin S Spiegelman BM The transcriptional coactivator PGC-1β drives the formation of oxidative type IIX fibers in skeletal muscle Cell Metab 2007 5 35 46 10.1016/j.cmet.2006.12.003 17189205

[B17] Bell AW Lipid metabolism in liver and selected tissues and in the whole body of ruminant animals Prog Lipid Res 1980 18 117 164 396532 10.1016/0163-7827(79)90013-4

[B18] Jeroch H Flachowsky G Weißbach F Futtermittelkunde 1993 Stuttgart: Gustav Fischer Verlag

[B19] National Research Council Guide for the care and use of laboratory animals 1985 Washington DC: National Institutes of Health Publication no. 85–23 (rev.)

[B20] Hämäläinen N Pette D The histochemical profiles of fast fiber types IIB, IID, and IIA in skeletal muscles of mouse, rat, and rabbit J Histochem Cytochem 1993 41 733 743 10.1177/41.5.8468455 8468455

[B21] Liu M Zhang D Wang X Zhang L Han J Yang M Xiao X Zhang Y Liu H Simultaneous quantification of niacin and its three main metabolites in human plasma by LC–MS/MS J Chromatogr B 2012 904 107 114 10.1016/j.jchromb.2012.07.030 22884475

[B22] Keller J Ringseis R Koc A Lukas I Kluge H Eder K Supplementation with l-carnitine downregulates genes of the ubiquitin proteasome system in the skeletal muscle and liver of piglets Animal 2012 6 70 78 10.1017/S1751731111001327 22436156

[B23] Ringseis R Mooren F Keller J Couturier A Wen G Hirche F Stangl GI Eder K Krüger K Regular endurance exercise improves the diminished hepatic carnitine status in mice fed a high-fat diet Mol Nutr Food Res 2011 55 Suppl 2 S193 202 21770048 10.1002/mnfr.201100040

[B24] Hemmings KM Parr T Daniel ZCTR Picard B Buttery PJ Brameld JM Examination of myosin heavy chain isoform expression in ovine skeletal muscles J Anim Sci 2009 87 3915 3922 10.2527/jas.2009-2067 19684280

[B25] Santschi DE Berthiaume R Matte JJ Mustafa AF Girard CL Fate of supplementary B-vitamins in the gastrointestinal tract of dairy cows J Dairy Sci 2005 88 2043 2054 10.3168/jds.S0022-0302(05)72881-2 15905435

[B26] Lin J Wu H Tarr PT Zhang C Wu Z Boss O Michael LF Puigserver P Isotani E Olson EN Lowell BB Bassel-Duby R Spiegelman BM Transcriptional co-activator PGC-1α drives the formation of slow-twitch muscle fibres Nature 2002 418 797 801 10.1038/nature00904 12181572

[B27] Lin J Handschin C Spiegelman BM Metabolic control through the PGC-1 family of transcription coactivators Cell Metab 2005 1 361 370 10.1016/j.cmet.2005.05.004 16054085

[B28] Yu S Reddy JK Transcription coactivators for peroxisome proliferator-activated receptors Biochim Biophys Acta 2007 1771 936 951 10.1016/j.bbalip.2007.01.008 17306620

[B29] Hagberg CE Falkevall A Wang X Larsson E Huusko J Nilsson I van Meeteren LA Samen E Lu L Vanwildemeersch M Klar J Genove G Pietras K Stone-Elander S Claesson-Welsh L Ylä-Herttuala S Lindahl P Eriksson U Vascular endothelial growth factor B controls endothelial fatty acid uptake Nature 2010 464 917 921 10.1038/nature08945 20228789

[B30] Monin G Mejenes-Quijano A Talmant A Sellier P Influence of breed and muscle metabolic type on muscle glycolytic potential and meat pH in pigs Meat Sci 1987 20 149 158 10.1016/0309-1740(87)90034-9 22056171

[B31] Fernandez X Meunier-Salaün M-C Ecolan P Glycogen depletion according to muscle and fibre types in response to dyadic encounters in pigs (Sus scrofa domesticus)–relationships with plasma epinephrine and aggressive behaviour Comp Biochem Physiol A Physiol 1994 109 869 879 10.1016/0300-9629(94)90234-8 7828029

[B32] Brewer MS Zhu LG Bidner B Meisinger DJ McKeith FK Measuring pork color: effects of bloom time, muscle, pH and relationship to instrumental parameters Meat Sci 2001 57 169 176 10.1016/S0309-1740(00)00089-9 22061360

[B33] Hambrecht E Eissen JJ Newman DJ Smits CHM Verstegen MWA den Hartog LA Preslaughter handling effects on pork quality and glycolytic potential in two muscles differing in fiber type composition J Anim Sci 2005 83 900 907 15753346 10.2527/2005.834900x

